# Tail‐dependent spatial synchrony arises from nonlinear driver–response relationships

**DOI:** 10.1111/ele.13991

**Published:** 2022-03-04

**Authors:** Jonathan A. Walter, Max C. N. Castorani, Tom W. Bell, Lawrence W. Sheppard, Kyle C. Cavanaugh, Daniel C. Reuman

**Affiliations:** ^1^ 2358 Department of Environmental Sciences University of Virginia Charlottesville Virginia USA; ^2^ 10627 Woods Hole Oceanographic Institution Woods Hole Massachusetts USA; ^3^ Department of Ecology and Evolutionary Biology and Center for Ecological Research and Kansas Biological Survey University of Kansas Lawrence Kansas USA; ^4^ Marine Biological Association of the United Kingdom Plymouth UK; ^5^ Department of Geography University of California, Los Angeles Los Angeles California USA

**Keywords:** copula, disturbance, giant kelp, *Macrocystis pyrifera*, nutrients, stability, synchrony, waves

## Abstract

Spatial synchrony may be tail‐dependent, that is, stronger when populations are abundant than scarce, or vice‐versa. Here, ‘tail‐dependent’ follows from distributions having a lower tail consisting of relatively low values and an upper tail of relatively high values. We present a general theory of how the distribution and correlation structure of an environmental driver translates into tail‐dependent spatial synchrony through a non‐linear response, and examine empirical evidence for theoretical predictions in giant kelp along the California coastline. In sheltered areas, kelp declines synchronously (lower‐tail dependence) when waves are relatively intense, because waves below a certain height do little damage to kelp. Conversely, in exposed areas, kelp is synchronised primarily by periods of calmness that cause shared recovery (upper‐tail dependence). We find evidence for geographies of tail dependence in synchrony, which helps structure regional population resilience: areas where population declines are asynchronous may be more resilient to disturbance because remnant populations facilitate reestablishment.

## Graphical abstract

Spatial synchrony may be tail‐dependent, i.e., stronger when populations are abundant than scarce, or vice‐versa. In sheltered areas, kelp declines synchronously (lower‐tail dependence) when waves are relatively intense, because waves below a certain height do little damage to kelp. Conversely, in exposed areas, kelp is synchronized primarily by periods of calmness that cause shared recovery (upper‐tail dependence).

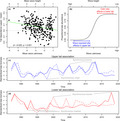

## INTRODUCTION

Spatial synchrony, the tendency for fluctuations to be correlated across locations, is a ubiquitous population dynamic phenomenon with important consequences for extinction risk and aggregate population variability (Liebhold et al., 2004). All else being equal, a more synchronous ensemble of populations faces higher extinction risk (Heino et al., 1998) and has a regional total population that is more temporally variable (Anderson et al., 2021) than a less synchronous ensemble. Recent studies of spatial synchrony have leveraged methodological advances to reveal new aspects of the phenomenon and strengthen inference into its mechanisms and consequences for ecological and socio‐environmental systems (Anderson et al., 2021; Ghosh et al., 2020b; Sheppard et al., 2016, 2019; Walter et al., 2017). For example, “geographies of synchrony” focus on spatial patterns, beyond the historically standard focus on how synchrony declines with increasing distance between locations, to reveal previously overlooked structural patterns and mechanisms that both cause synchrony and modify its strength (Larsen et al., 2021; Walter et al., 2017).

**FIGURE 1:**
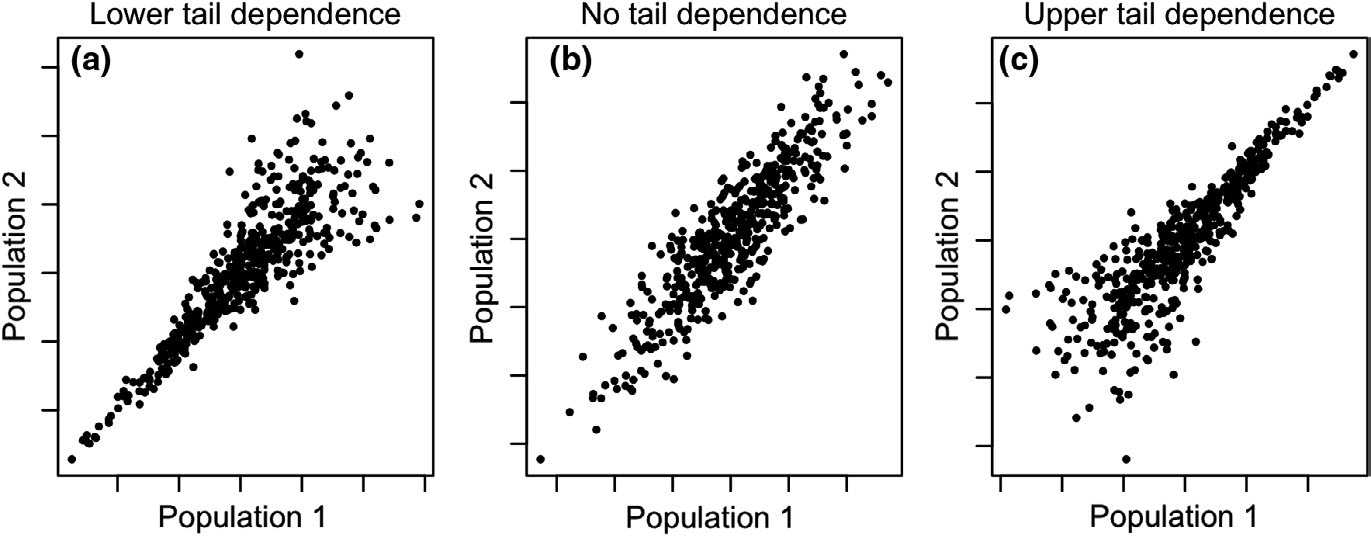
Illustration of relationships with (a) lower‐tail dependence (asymmetric association), (b) no tail asymmetry, and (c) upper‐tail dependence in three hypothetical population pairs with the same overall strength of association (Spearman rank correlation = 0.8)

Another newly introduced aspect of synchrony with a potential to reveal novel patterns and strengthen mechanistic understanding is the tendency for the strength of relatedness between two variables to differ between the upper and lower portions of a variable's distribution (Figure 1; Ghosh et al., 2020a,b,c), in this study called ‘tail dependence’. Here, ‘tails’ refers to the upper or lower portions of a variable's distribution. Tail‐dependent associations between ecological and environmental variables are common (Ghosh et al., 2020a), including in the context of synchrony among interacting species in a community (Ghosh et al., 2020b, 2021). However, tail dependence in spatial synchrony has been little studied, so its prevalence, mechanisms, and consequences in empirical populations are not yet well understood. In principle, tail‐dependent spatial synchrony should have substantial implications for extinction risk (Ghosh et al., 2020c). An ensemble of populations exhibiting stronger inter‐population associations in the upper tails of population distributions will have spatially synchronised population booms, leading to widespread periods of high abundance, but populations exhibiting stronger associations in their lower tails will experience synchronised crashes. Synchronised population crashes that inhibit dispersal‐mediated rescue effects are a known consequence of synchrony, generally (Heino et al., 1998), and for populations with strong lower tail association the extinction risk will be greater than an ensemble of populations with equal spatial synchrony and no asymmetries of tail association (Ghosh et al., 2020c).

Throughout this manuscript, ‘tail dependence’ refers to a quantity differing as it manifests in the upper and lower portions of one or more distributions, for example, synchrony can be called tail‐dependent if it is stronger in the lower tails of the distributions of the synchronised quantities than in the upper tails, or vice versa; whereas ‘tail association’ just refers to associations between variables in one or both of the tails. These terminological choices differ slightly from previous related work (see *Discussion*). We emphasise that this study focuses on the tails of distributions of abundance and of values of environmental drivers, not on the tails of dispersal kernels, a different sort of tail familiar to many ecologists. The tail properties of dispersal kernels (e.g. whether they are `fat’ or `thin’) likely influence spatial synchrony but are not the focus of this study. Furthermore, whereas the ‘lower tail’ may sometimes be understood as the absolute extreme lower portion of a distribution, we instead use the term to refer, more loosely, to the lower half of the distribution; analogously for the upper tail.

A likely common mechanism of tail‐dependent relationships arises from nonlinear, threshold‐like relationships between an ecological variable and an environmental driver (Ghosh et al., 2020a). Many examples of such relationships have been observed. For example, plant seed germination may be a decreasing sigmoid function of environmental stressors (Chauhan & Johnson, 2009). Similarly, growth rates of phytoplankton exhibit threshold‐like responses to increasing nutrient levels as a particular nutrient moves from limiting to abundant (Interlandi & Kilham, 2001). In threshold‐like relationships, values of the environmental driver in the upper or lower extremes have little relationship to the ecological response, but fluctuations near and across the threshold have large effects. This can produce tail dependence in the relationship between driver and response, with the direction (i.e. stronger relationship in either the upper or lower tail) depending on whether mean environmental conditions are below or above the threshold. Since environmental drivers tend to be spatially autocorrelated (Di Cecco & Gouhier, 2018; Koenig, 2002), we expect that tail dependence in relationships between drivers and ecological variables may produce tail‐dependence in spatial synchrony.

Here, we demonstrate how tail‐dependent spatial synchrony can arise from threshold‐like relationships between ecological responses and environmental drivers using a general theoretical model; and we also analyse an empirical instance of our theoretical ideas in populations of giant kelp (*Macrocystis pyrifera*). Giant kelp is a superb organism for studying spatiotemporal dynamics (Bell et al., 2015; Castorani et al., 2015, 2017; Cavanaugh et al., 2013), as well as being the foundation species of productive (Castorani et al., 2021) and diverse coastal ecosystems (Castorani et al., 2018). We show that giant kelp exhibits geographically variable tail dependence in spatial synchrony. Consistent with our theory, spatial variability in tail dependence of kelp synchrony can be explained by the nonlinear relationship between wave intensity and damage to kelp, and geographical variability in mean wave intensity. Areas where spatial synchrony tends to be stronger in the lower tails of kelp distributions (i.e. where crashes in kelp abundance are more synchronous than booms) are predicted to be more vulnerable to extirpation. Our results provide new information about an ecosystem of great interest to coastal ecologists and resource managers; but perhaps more importantly, because of the generality of our theory and the commonness of its key ingredients, our findings also demonstrate a new phenomenon and mechanism which may be present but previously unrecognised in many other systems.

## SUMMARY OF THEORY AND EXAMPLE

**FIGURE 2:**
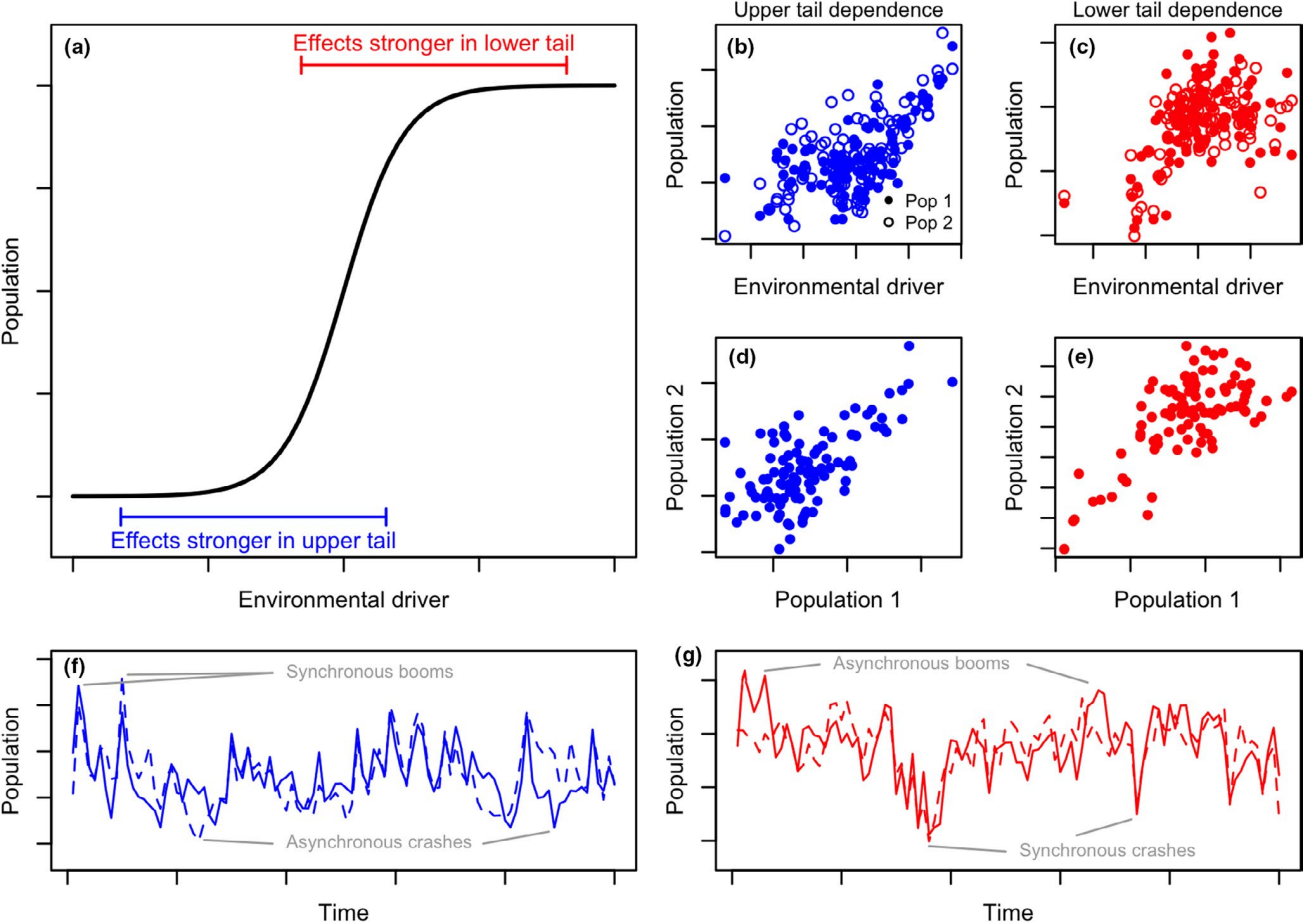
Example illustrating how threshold‐like relationships between population dynamics and an environmental driver can produce tail‐dependent spatial synchrony. The modelling approach is summarised in the text, with mathematical details in Supplementary Materials S1. (a) A sigmoid relationship is assumed between an environmental variable and its effects on populations. Locations for which mean environmental conditions are below the threshold (range of environmental driver shown by blue bar) experience stronger population effects in the upper tail, whereas locations for which mean environmental conditions are above the threshold (range shown by red bar) experience stronger population effects in the lower tail. (b‐c) Asymmetric tail associations between populations and the driver variable arise from the model. (d‐e) Tail‐dependent spatial synchrony then occurs between populations having shared responses to the driver. (f‐g) Time series for populations in (d‐e). Note that the populations with stronger upper‐tail association (d) show a tendency towards synchronised booms with less synchronous crashes (f), while the populations with stronger lower‐tail association (e) show a tendency towards synchronised crashes and less synchronous booms (g)

We here summarise our general theory and an example. Mathematical details are in Supplementary Material S1. We begin by describing intuition behind the theory. When there is a threshold‐like (e.g. sigmoid) relationship between an environmental driver and its effects on a population, environmental variations in the upper or lower extremes have limited population effects because a threshold or sigmoid function is flat or nearly flat in its extremes. However, a population will be much more sensitive to environmental variation across the threshold. For a given location, environmental conditions may be above or below the threshold, on average, but may vary in one tail across the threshold; thus environmental variation in that tail, but not in the other tail, has large population influence (Figure 2a). Average environments below the threshold lead to environment‐population associations that are stronger in the upper tails (Figure 2b), whereas average environments above the threshold lead to associations that are stronger in the lower tails (Figure 2c). When, as is often the case, the driver is spatially autocorrelated, it imparts tail‐dependent spatial synchrony to populations (Figure 2d,e). A set of populations with stronger synchrony in their upper tails will experience synchronised booms and less synchronised crashes, whereas populations with stronger synchrony in the lower tails will experience the reverse (Figure 2f,g).

Our general model is $N_{i}\left(t+1\right)=N_{i}\left(t\right)\lambda \left(N_{i}\left(t\right)\right)\exp\left(e_{s}\left(t\right)+e_{l,i}\left(t\right)\right)$ for i=1,2, where $N_{i}\left(t\right)$ is population density at location i and time t, λ(N) is a density‐dependent growth rate which declines monotonically to zero as N gets large, $e_{s}\left(t\right)$ is a spatially synchronous environmental effect on growth rates, and $e_{l,i}\left(t\right)$ represents local‐noise effects. We let $e_{s}\left(t\right)=f\left(\delta \left(t\right)\right)$, where δ(t) is a spatially synchronous environmental variable and f, a sigmoid function (Figure 2a, equation S2), represents the nature of the influence of δ on growth rates. In Supplementary Material S1, we show that, quite generally under this model setup, when the distribution of δ is positioned below the threshold‐like portion of f, but with its upper tail overlapping the threshold, the populations $N_{1}\left(t\right)$ and $N_{2}\left(t\right)$ show stronger upper‐ than lower‐tail association; whereas when δ is positioned somewhat above the threshold, the populations show stronger lower‐ than upper‐tail association. Because λ is arbitrary, conclusions are fairly general, though of course not universal. The specific case of our model that was used for Figure 2 is in Supplementary Material S2.

Our theory leads to several hypotheses about real systems. If the effects of an environmental driver on population dynamics are threshold‐like (such as the sigmoid function considered here), and if the distribution of values of the environmental driver is, in some locales, on the lower shoulder and, in other locales, on the upper shoulder of this relationship, then one expects: (A) geographic variation in tail dependence of population synchrony; (B) geographic variation in tail dependence of the relationship between populations and their local driver; and (C) correlation between the patterns of variation in A and B. We tested whether the above predictions describe patterns of tail dependence of kelp synchrony and causes thereof.

## METHODS

### Study system

Giant kelp is a globally distributed species that forms a conspicuous monospecific surface canopy that makes it amenable to remote measurement over large areas and decadal time‐spans (Bell et al., 2020; Cavanaugh et al., 2011). Its rapid growth and reproduction also allow for the examination of numerous generational cycles relative to what is possible in many terrestrial ecosystems (Reed et al., 2008). Throughout coastal California, USA, giant kelp biomass is synchronous on two characteristic spatial scales reflecting complex drivers of giant kelp abundance (Cavanaugh et al., 2013). Over distances from tens of meters to ≈1.5 km, spatial synchrony declines rapidly with distance, approximately matching the spatial scale of recruitment and spatial autocorrelation in abundances of grazing sea urchins (Cavanaugh et al., 2013). Over much longer distances, >100 km, synchrony declines more slowly with distance, similar to the characteristic spatial scales of regional‐scale oceanographic patterns in environmental drivers including nutrient concentrations and destructive storm‐driven waves (Cavanaugh et al., 2013). Sub‐regional spatial structure in giant kelp dynamics can also be attributed to differences in wave exposure (Cavanaugh et al., 2011) and other evidence (Bell et al., 2015) supports the hypothesis that differences in wave height are a major source of spatial structure in giant kelp dynamics on the California coast. North of Point Conception, excepting small protected embayments, the California coast is exposed to strong winter waves that tend to cause seasonal declines in kelp canopy biomass. Rapid growth enables kelp canopy recovery during the spring and summer, provided adequate nutrient availability. The coast south of Point Conception is relatively sheltered from waves because of the orientation of the coastline relative to northwest swells and protection by the California Channel Islands.

### Datasets

We developed 33‐year time series (1987 through 2019) of annual mean giant kelp canopy wet biomass (kg) for coastline segments spanning southern and central California, USA by calibrating satellite remote sensing data to field observations. Methods are described in detail in Cavanaugh et al. (2011) and Bell et al. (2020), and briefly summarised here. The fraction of kelp cover in Landsat images (30 m pixels) was estimated using a spectral unmixing algorithm with kelp and water endmembers. An empirical relationship was used to convert the satellite‐derived kelp cover data to kelp biomass. The data were spatially aggregated to 500 m segments of mainland coastline, and were temporally aggregated to quarter and then to year. Kelp forests along the Channel Islands were not considered in this study because of limited data availability.

We selected coastline segments where kelp was absent (zero or no data) for no more than 3 years during the study period to focus on sites where kelp was largely persistent throughout the study period. Of the 437 coastline segments for which kelp was present in at least one year, 361 (83%) were selected for analysis.

We obtained data on three key environmental drivers of giant kelp dynamics: wave intensity, seawater nitrate concentration, and the North Pacific Gyre Oscillation (NPGO) index. Annual average maximum significant wave heights were obtained from the CDIP MOP v1.1 model (http://cdip.ucsd.edu/MOP_v1.1/) and aggregated to 500 m coastline segments. Briefly, the model combines hourly empirical measurements of significant wave height and direction (from the U.S. National Buoy Data Center, NBDC) with swell propagation and swell hindcast models (Hanson et al., 2009; O'Reilly et al., 2016; Wingeart et al., 2002). We computed the annual average surface seawater nitrate concentration for each 500 m segment using established empirical relationships between nitrate concentration and sea surface temperature (Palacios et al., 2013; Snyder et al., 2020; Zimmerman & Kremer, 1984). Sea surface temperature estimates were obtained from Advanced Very High Resolution Radiometer satellite imagery (Banzon et al., 2016; Reynolds et al., 2007). Detailed methods for computation of the wave intensity and seawater nitrate data are given in Bell et al. (2015). Values of the NPGO index were obtained from Di Lorenzo et al. (2008). Positive values of the NPGO index correspond to strengthening of wind‐driven upwelling and enhanced nearshore nutrient concentrations (Di Lorenzo et al., 2008; Pennington & Chavez, 2018). Note that unlike the wave and nitrate data, the NPGO index does not vary spatially, although its effect on giant kelp does (Bell et al., 2015).

Because our main statistical procedure (see *Analyses* below) is valid for positively correlated variables only and the expected correlation between wave intensity and kelp biomass is negative (increases in wave intensity cause decreases in kelp biomass), we transformed the wave height variable into ``wave calmness" by multiplying by −1. Prior to analysis, we removed linear temporal trends from all time series.

### Analyses

We assessed tail dependence in giant kelp spatial synchrony and in the relationship between kelp biomass and environmental drivers using the partial Spearman correlation (Ghosh et al., 2020a,b,c; 2021). Given two bounds $0\leq b_{l}<b_{u}\leq 1$ (l stands for `lower’ and u for ‘upper’), for two positively correlated variables, the partial Spearman correlation is the portion of the standard Spearman rank correlation which arises because of the range of quantiles in the two variables bounded by $b_{l}$ and $b_{u}$ (see Supplementary Materials S3 for mathematical details). We computed the partial Spearman correlation for the lower portions of the two distributions ($b_{l}=0$ and $b_{u}=0.5$) and for the upper portions ($b_{l}=0.5$ and $b_{u}=1$), separately, and took the difference (upper ‐ lower) as a metric of the strength of tail dependence (Ghosh et al., 2020b). Similar to (Ghosh et al., 2020b,c), we chose values for $b_{l}$ and $b_{u}$ that include middle portions of the distribution, not strictly the extremes, so that there were sufficient data to quantify association strength. Positive values of tail dependence strength indicate stronger association in the upper tails, and negative values indicate stronger association in the lower tails. To study tail dependence in kelp biomass spatial synchrony, partial Spearman correlations and tail dependence strengths were computed between all pairs of coastline segments. To study tail dependence in the relationship between kelp biomass and environmental drivers, we computed partial Spearman correlations and tail dependence strengths between kelp biomass and, respectively, wave calmness, nitrate concentration, and the NPGO index.

To help answer whether giant kelp exhibits consistent or geographically dependent tail dependence in spatial synchrony (prediction A from *Theory*), we first asked whether distance‐decay in synchrony depended on whether we considered lower or upper tails of kelp biomass. We fit non‐parametric spatial covariance functions (Bjørnstad & Falck, 2001), using the partial Spearman correlation as the synchrony variable, to all sites and to the southern and central California coasts, separately. Point Conception was used to separate southern from central California because preliminary analyses showed that kelp forests north of Point Conception tended to be more synchronous with each other and less synchronous with sites south of Point Conception, and vice‐versa. A standard bootstrapping procedure (Bjørnstad & Falck, 2001) was used to generate 95% confidence bands around non‐parametric spatial covariance functions.

Additionally, we investigated matrices of pairwise synchrony (partial Spearman correlation) for the upper and lower tails, as well as the element‐wise difference between these matrices, that is, the tail dependence strength in synchrony between each pair of sites. We used surrogate‐based statistical tests to determine whether the overall strength of tail association, and its spatial structure, were significantly different from a null hypothesis of no tail association. Following the methods described in detail in section S9 of the Appendices of Ghosh et al. (2020a), we created 1000 synthetic kelp datasets that had the same number of years and locations, and approximately the same Spearman correlations between all pairs of sites as the original data, but with no asymmetries of tail association between any pairs of sites (to within sampling variation). For each surrogate dataset, we then computed two statistics and compared their distributions to the values of the same statistics for the empirical data. The first statistic was the sum, across all pairs of sites, of tail dependence strengths. The empirical value was considered to diverge significantly from the null distribution of surrogate values if its quantile in the surrogate distribution was less than 0.025 or greater than 0.975. The second statistic, corresponding to a test for geographically dependent asymmetry in tail association, was equal to $1‐\mathrm{cor}(M_{u},M_{l})$, where `cor’ is the Pearson correlation and $M_{u}$ and $M_{l}$ are the synchrony matrices for the upper and lower tails. A large value of this statistic corresponds to relatively uncorrelated entries in the $M_{u}$ and $M_{l}$ matrices, that is, to different geographies of synchrony in the upper and lower tails of kelp distributions. This in turn indicates spatial structure in the tail dependence of synchrony. Surrogate datasets have relatively small values of this statistic, since the surrogate procedure eliminates asymmetries of tail association, so corresponding entries of $M_{u}$ and $M_{l}$ should be about the same, except for sampling variation. A value of the statistic computed on real data was considered significant if its quantile relative to surrogates was >0.95. The p‐value associated with the test was 1 minus this quantile.

**FIGURE 3:**
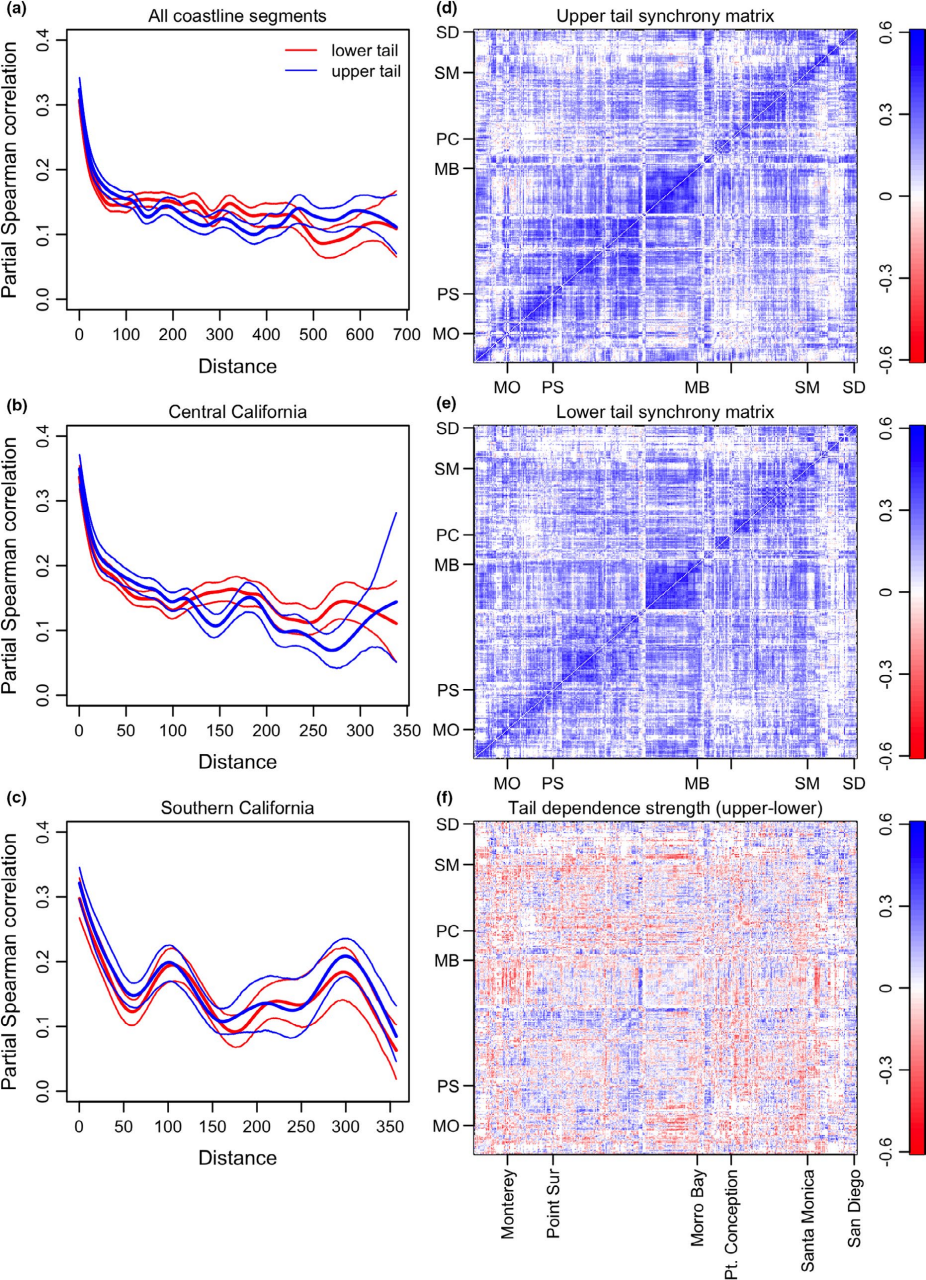
(a‐c) Distance decay in upper‐ and lower‐tail spatial synchrony, considering (a) all coastline segments; (b) central California; and (c) southern California. Central and southern California were divided at Point Conception (see Figure 4). (d‐f) Matrix depictions of tail dependent spatial synchrony in giant kelp. Matrices are indexed by coastline segment, in along‐shore order beginning from the northernmost site near Monterey, California. Panels (d) and (e) give the synchrony matrix (partial Spearman correlation) for upper and lower tails, respectively; (f) gives the tail dependence strength (panel d minus panel e)

To assess whether there is geographic variation in tail dependence of the relationship between environmental drivers and kelp populations (prediction B from *Theory*), and to test whether tail dependence in kelp spatial synchrony could be explained by tail dependence in the relationship between kelp abundance and environmental drivers (prediction C from *Theory*), we used generalised least squares spatial multiple regression. We took the average strength of tail dependence in kelp synchrony between each coastline segment and those within 25 km of it to associate a single value with each site, similar to the approach of (Defriez & Reuman, 2017a,b). Similarly, we averaged the strength of tail dependence for the relationship between kelp biomass and each of wave calmness, nitrate concentration, and the NPGO index (separately) within 25 km of each site. Twenty‐five km was chosen as a threshold distance for averaging because synchrony tended to decline rapidly over distances up to ≈25 km (Figure 3). Parallel analyses assessing the robustness of our results to distance thresholds ranging from 10 to 200 km are presented in Tables S1‐S4. We tested for the effects of all three predictors simultaneously in a multiple regression framework fit with generalised least squares, considering model error to have spatial correlation that declines as a negative exponential function of distance. To aid in interpreting results of this analysis, we subsequently used spatial multiple regression fit with generalised least squares to test for a relationship between the coastline segment mean wave calmness and tail association in the correlation between kelp biomass and wave calmness, while controlling for spatial autocorrelation using a negatively exponentially correlated error term. We did not explicitly consider the role of dispersal in structuring kelp synchrony because we focused on synchrony over distances substantially larger than kelp spores typically disperse (Gaylord et al., 2004, 2006).

Additional details on spatial regression statistical models and tests of conformity with model assumptions are in Supplementary Material S4. Statistical models were fit using generalised least squares regression in the ‘nlme’ R package (Pinheiro et al., 2021). All analyses were conducted in R version 4.0.3 (R Core Team, 2020).

## RESULTS

### Tail dependence in giant kelp spatial synchrony

Confirming theoretical prediction A (*Theory*), that there would be geographical variation in tail dependence of population synchrony, kelp synchrony exhibited geographically structured tail dependence. Kelp synchrony was not consistently greater in the upper or lower tails, as a function of distance, over the whole coast or considering only central or southern California (Figure 3a‐c). Although they were sometimes divergent, in most cases the 95% confidence bands of non‐parametric spatial covariance functions remained overlapping. Supporting these results, the total, across all pairs of locations, tail dependence strength in synchrony was not statistically different from zero (i.e. no tail association). The quantile of the empirical statistic for this test relative to surrogates was 0.45, which is neither <0.025 nor >0.975.

However, some parts of the coast showed strong differences (i.e. up to ≈±0.5) in strength of association between upper and lower tails, and a matrix describing tail dependence in pairwise spatial synchrony reveals stretches of coastline where upper or lower tail associations are prevalent (deep blue and red areas in Figure 3f). Our test for geographic structure in tail dependence (see *Methods*) was highly significant(p<0.0001).

**FIGURE 4:**
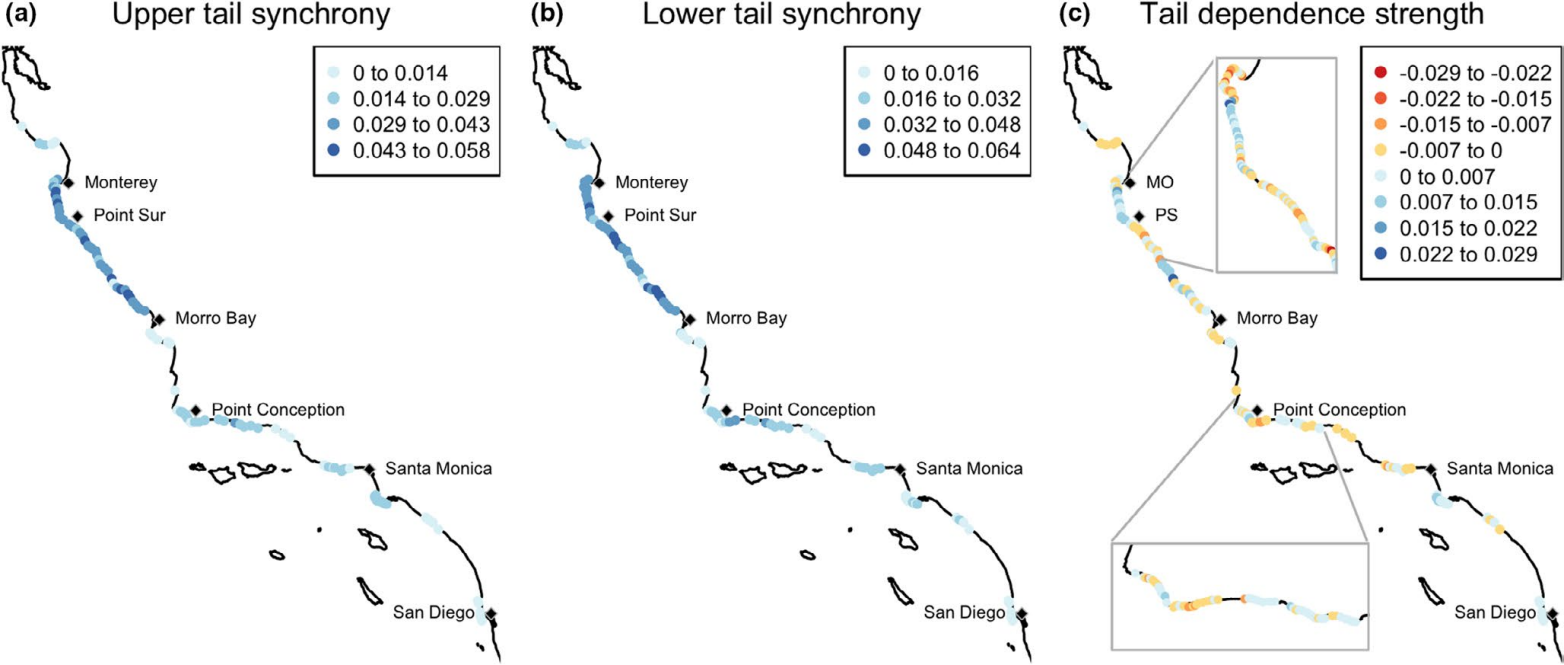
Maps of tail‐specific spatial synchrony and tail dependence strength. Values are averaged over pairs of sites within 25 km. To reduce overlap between icons for nearby shoreline segments, overview maps are thinned by displaying every fifth coastline segment in high data‐density areas and by displaying every other coastline segment in low data‐density areas. Every coastline segment is displayed in inset maps. MO =Monterey, PS =Point Sur

Mapping mean tail‐specific synchrony and tail dependence among coastline segment pairs within 25 km corroborated that tail‐specific synchrony and tail association strength were heterogeneous across the central and southern California coasts (Figure 4). Synchrony tended to be greatest along the central coast between Morro Bay and Monterey (Figure 4a, b). This region also tended to show a large magnitude and great variety in tail dependence strength, including both substantially positive and substantially negative values. Examples of site pairs exhibiting lower and upper tail dependence, corresponding to empirical examples of Figure 2b‐c, are shown in Figure S2.

### Environmental drivers of tail dependence

**FIGURE 5:**
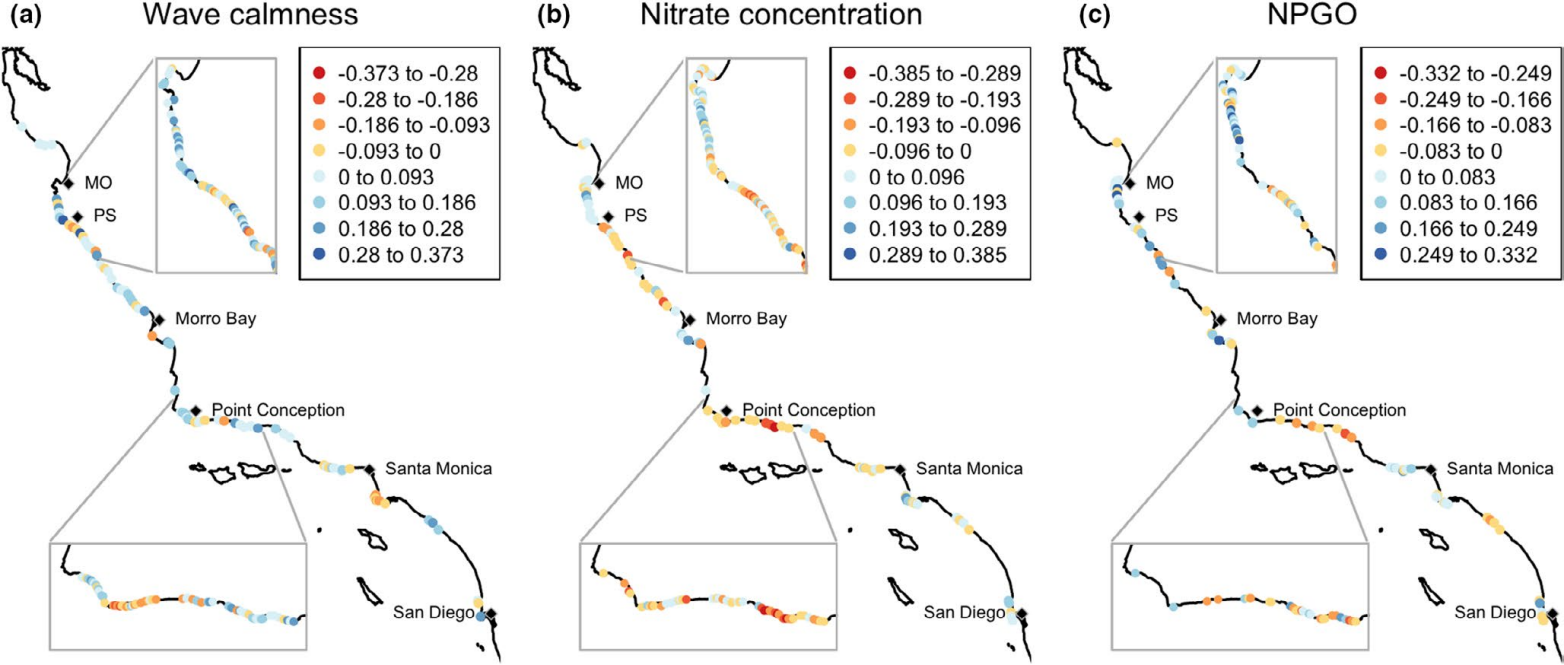
Maps of tail dependence strength in correlations between giant kelp biomass and environmental drivers. To reduce overlap between icons for nearby shoreline segments, overview maps are thinned by displaying every fifth coastline segment in high data‐density areas and by displaying every other coastline segment in low data‐density areas. Every coastline segment is displayed in inset maps. MO = Monterey, PS = Point Sur

Similar to tail dependence in giant kelp spatial synchrony, strength of tail dependence in the correlation between giant kelp biomass and environmental drivers varied widely across the study area (Figure 5). This supports theoretical prediction B, that tail dependence in the relationship between populations and their local driver will vary spatially. There were nearly the same number of coastline segments with stronger lower‐tail association as with stronger upper‐tail association for nitrate concentration (median =0.004) and the NPGO (median =0.009). The relationship between kelp biomass and wave calmness showed a slight tendency for more coastline segments to have greater upper‐tail association (median =0.060). Mapping tail dependence strengths revealed stretches of coastline where upper (positive values) or lower (negative values) tail dependence predominates (Figure 5), and there were visual similarities between the map of tail dependence in giant kelp synchrony (Figure 4c) and tail dependence in driver relationships (Figure 5). For example, between Point Conception and Santa Monica, the pattern of lower (yellow to red) and upper (light to dark blue) tail dependence is similar between kelp spatial synchrony (Figure 4c) and the relationship between wave calmness and kelp biomass (Figure 5a). Examples of sites exhibiting lower and upper tail dependence, corresponding to empirical examples of Figure 2d‐e, are shown in Figure S2.

Formally testing for statistical relationships between tail dependence in kelp synchrony and tail dependence in relationships with wave calmness, nitrate concentration, and the NPGO in a spatial multiple regression supported theoretical prediction C, that geographical patterns in tail dependence in spatial synchrony and geographical patterns in tail dependence in the relationship between populations and local drivers would be correlated. Tail dependence in the relationship between kelp and wave calmness was a highly significant predictor (β=0.11±SE=0.025,p=0.0002) of tail dependence in giant kelp spatial synchrony (Table S2). The effects of nitrate tail association (β=‐0.0040±0.030,p=0.89) and NPGO (β=‐0.016±0.027,p=0.56) were non‐significant. The statistical significance and effect direction were consistent using 10 km, 25 km, 50 km, 100 km, and 200 km distance thresholds (Tables S1‐S5).

**FIGURE 6:**
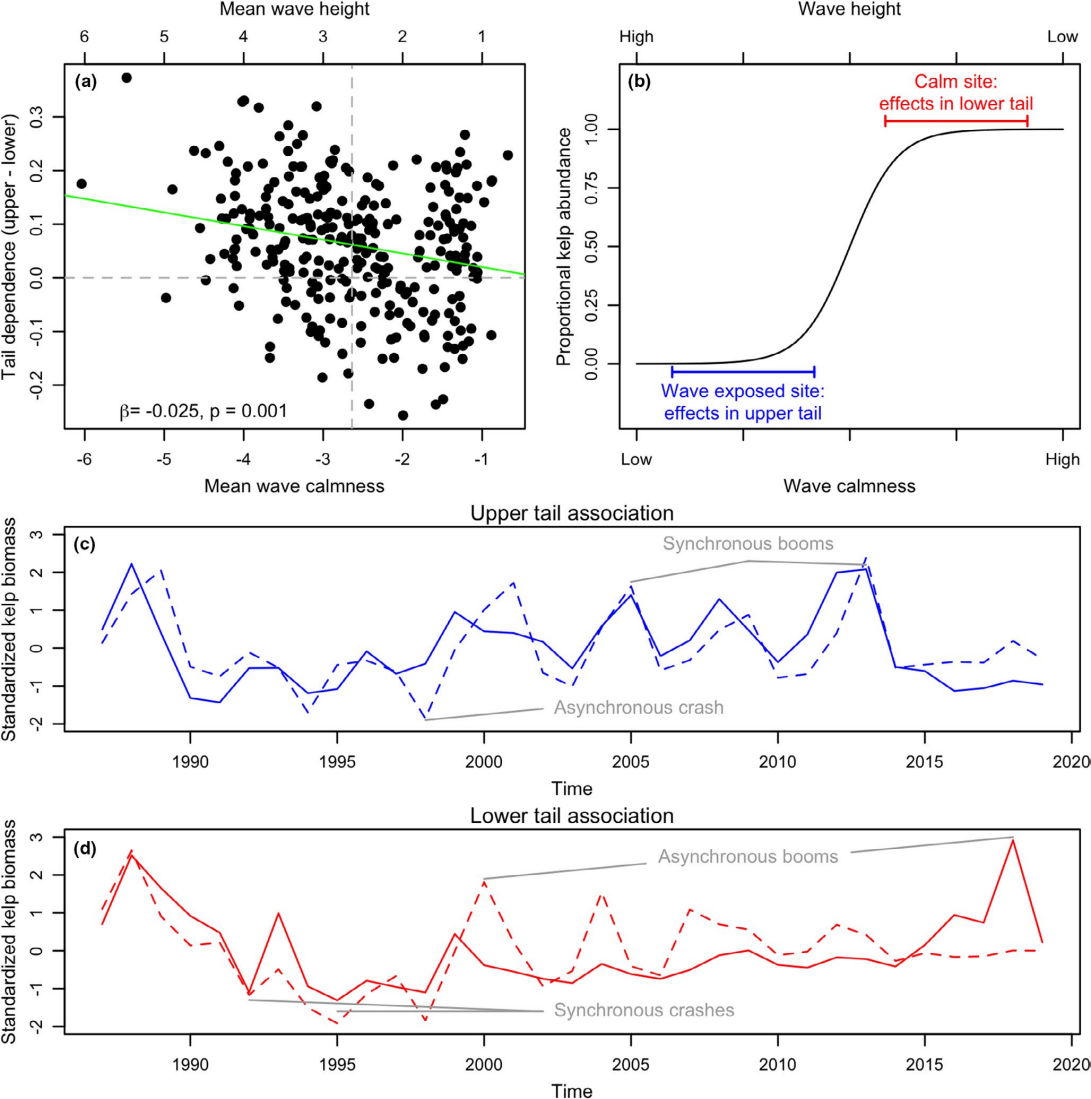
(a) Tail dependence in the relationship between giant kelp biomass and wave calmness is negatively related to (β=‐0.025, p=0.001 using linear modeling techniques of *Methods* that account for spatial autocorrelation) mean wave calmness such that stronger upper tail dependence is more prevalent in areas with low average wave calmness (higher average wave height). The horizontal dashed line indicates tail dependence strength = 0; the vertical dashed line denotes the median site mean wave calmness. (b) Idealised sigmoid relationship between wave calmness and persistence of kelp biomass illustrating how tail dependence can depend on whether a coastline segment is, on average, calm or wave exposed. Blue and red horizontal bars indicate the range of wave calmness experienced at, respectively, theoretical wave‐exposed and calm sites. (c, d) Selected kelp biomass time series illustrating how a pair of coastline segments can be synchronised (c) in the upper tail, resulting in synchronous booms and less synchronous crashes, or (d) synchronised in the lower tail, resulting in synchronous crashes and less synchronous booms

Further confirming C from *Theory* and our overall proposed mechanism, there is also a significant negative relationship between the average wave calmness for a site and tail dependence in the relationship between kelp biomass and wave calmness (β=‐0.025, p=0.001; Figure 6a).

## DISCUSSION

Tail‐dependent spatial synchrony can arise from threshold‐like relationships between environmental drivers and driven ecological variables, in theory and in empirical data. Using methods recently introduced to ecology (Ghosh et al., 2020b,c), we have shown empirically for what we believe to be the first time that there is a geography of tail dependence in spatial synchrony; and we have documented a likely mechanism. Specifically, there is a geography of tail dependence in kelp spatial synchrony that is partly explained by geographic patterns of tail dependence in the association between waves and kelp biomass. That is, across the southern and central California coasts, whether spatial synchrony in giant kelp biomass is stronger in the upper or lower tail of the distribution varies, and this variation is due in part to whether the relationship between waves and kelp biomass along a stretch of coastline tends to be stronger in the upper or lower tail. This, in turn, depends on the average wave intensity at a site because the relationship between wave intensity and wave effects on kelp is nonlinear: above a certain level, increasing wave intensity has little effect because kelp has already been severely damaged; whereas below a certain level, decreasing wave intensity has little effect because waves are already small enough to cause no damage (Figure 6; Bell et al., 2015; Cavanaugh et al., 2011; Seymour et al., 1989; Utter & Denny, 1996). Therefore, areas with high wave intensity (low wave calmness) tended to show stronger upper‐tail associations between waves and kelp biomass, whereas areas with low wave intensity (high wave calmness) tended to show stronger lower‐tail associations.

The mechanism for tail‐dependent spatial synchrony demonstrated by this study is likely quite general, for reasons including and beyond the flexibility of our theoretical model (Supplementary Material S1). While a threshold‐like or sigmoid relationship is the simplest functional form that can produce switching between upper‐ and lower‐tail dependence, any nonlinear relationship can produce tail dependence. For example, an increasing saturating function produces lower‐tail dependence with strength depending on the mean and variance of fluctuations in the environmental driver. Arguably, a large majority of population effects of environmental drivers have sigmoid or saturating shapes, even if they are approximately linear over some ranges of the driver. Nonlinear, threshold‐like relationships have been observed in many ecological phenomena (e.g. Chauhan & Johnson, 2009; Interlandi & Kilham, 2001; Utter & Denny, 1996). Increasing saturating functions are likely even more common than threshold‐like relationships because they arise from limitations in fundamental biochemical processes (Michaelis & Menten, 1913). As environmental variables such as temperature and rainfall become more variable, distributions may become more likely to overlap with shoulders on sigmoidal or threshold‐like response curves, producing new asymmetric tail associations in driver‐population relationships and in spatial synchrony.

One common mechanism of spatial synchrony not explicitly considered in our study is dispersal. This choice was made for theoretical simplicity and because our empirical analysis considered spatial scales (0.5 to 700km) substantially larger than typical giant kelp spore dispersal distances (meters to perhaps a few kilometers; Gaylord et al., 2004, 2006; Reed et al., 2006), minimising the importance of dispersal to observed patterns. Note, however, that dispersal may be important to synchrony at spatial scales exceeding the scale of dispersal when the dispersal rate is much greater than the strength of density regulation (Lande et al., 1999). The mechanism of tail‐dependent spatial synchrony described here can operate across spatial scales up to the range of spatial autocorrelation in the environmental driver, which is often orders of magnitude greater than the range of dispersal. Even at scales where dispersal is relevant, we do not expect dispersal to alter the tail dependence of spatial synchrony if dispersal is density‐independent. However, we predict that density‐dependent dispersal could generate tail‐dependent spatial synchrony. How multiple mechanisms that independently would impart tail‐dependent spatial synchrony interact is an opportunity for future research.

Tail‐dependent spatial synchrony represents a new extension of spatial synchrony's general negative implications for the stability of population ensembles (Anderson et al., 2021; Heino et al., 1998; Schindler et al., 2015). Because of the implications of tail‐dependent spatial synchrony for the tendency towards synchronised booms versus synchronised crashes, understanding the nature of tail associations and how they vary spatially can help explain patterns of temporal stability. In giant kelp, we found that areas tending towards stronger lower‐tail association in kelp synchrony should experience synchronised crashes with severe losses over coastline stretches spanning tens of kilometers. Conversely, areas tending towards stronger upper‐tail associations in kelp synchrony should experience synchronised periods of high biomass (i.e. booms) but with crashes tending to be less synchronous. Given the importance of propagule (spore) dispersal from nearby kelp forests for re‐establishment following extirpation (Castorani et al., 2015, 2017), our results suggest that areas where kelp has stronger synchrony in the upper tails could be especially resilient to perturbations, all else being equal. However, resilience could be strained if climate change makes large‐scale crashes more common because giant kelp spore dispersal declines rapidly with distance, with maximum recruitment distances generally < 10 km (Reed et al., 2006). Extreme oceanographic events such as the 1997–1998 El Niño and 2014–2016 ‘warm blob’ marine heatwave caused substantial region‐wide declines in giant kelp (Cavanaugh et al., 2019; Edwards, 2004). Large‐scale extreme events such as these, or other situations where multiple stressors impact kelp populations (McPherson et al., 2021; Rogers‐Bennett & Catton, 2019), may overwhelm local heterogeneity‐‐‐here because of differences in coastline orientation that shelter populations from waves‐‐‐thereby limiting recolonisation via spore dispersal from sheltered refugia (Castorani et al., 2015, 2017).

In addition to waves, we considered whether tail dependence in the relationship between giant kelp and, respectively, seawater nitrate concentrations and the NPGO explained tail dependence in giant kelp synchrony. Although these are likely drivers of overall spatial synchrony of giant kelp (Bell et al., 2015; Cavanaugh et al., 2013), they did not explain geographical patterns of tail‐dependent spatial synchrony in giant kelp. The likely reason is that the relationships between giant kelp and these two variables are not sigmoid or threshold‐like (Bell et al., 2015). Thus, our empirical study of giant kelp supports our general theory through both a positive example and two negative examples in which the phenomenon was not observed when the conditions of the theory were not met.

What we call tail dependence in this manuscript has been called ‘asymmetric tail association’ or simply ‘tail association’ in other studies (Ghosh et al., 2020a,b,c; 2021). The terminology ‘lower‐tail dependence’ or ‘upper‐tail dependence’ was used in early work (Ghosh et al., 2020a) to describe associations between variables in the corresponding tails, but this terminology was later abandoned in favour of ‘lower‐tail association’ and ‘upper‐tail association’ (Ghosh et al., 2020b, 2021), because the earlier terminology could be construed to imply a direct causal dependence between the variables, which need not be the case. We found utility in distinguishing between tail association, which as used here does not necessarily imply difference or asymmetry between the tails, and tail dependence, which does; we preferred the brevity of tail dependence to asymmetric tail association, but note that these refer to the same concept. Our current use of ‘dependence’ refers to dependence of an association on distributional tail, and does not in and of itself imply any direct causal relationship between the variables.

Here, we introduced a general theoretical framework for a mechanism of tail‐dependent spatial synchrony and provided empirical evidence of its manifestation in synchrony of giant kelp. Although we provide the first empirical evidence for geographies of tail dependence in spatial synchrony, we predict that tail dependence in spatial synchrony, and geographies thereof, may be quite common, for several reasons. First, tail dependence is common in ecological relationships (Ghosh et al., 2020a). Second, and related, sigmoidal/threshold/nonlinear relationships between drivers and population effects are ubiquitous. Third, it is common for distributions of the local values of an environmental variable to differ across the geographic range of a species, and for these distributions to therefore fall on different parts of the response curve. Thus all the ingredients of our theory and of our empirical example are common. The approaches used herein provide an example of how future studies can quantify tail dependence in spatial synchrony and infer its likely mechanisms. Related methods based on synchrony networks or matrix regression (Walter et al., 2017) would also be appropriate. Tail dependence affects stability (Ghosh et al., 2021) and extinction risk (Ghosh et al., 2020c), over and above the implications of synchrony itself for those same factors, and has particular value for understanding the dynamics of catastrophes and recoveries. Consequently, developing a more complete understanding of how tail dependence manifests in synchronous ecological phenomena is important to understanding mechanisms of synchrony and the stability of ecological systems, two longstanding challenges.

## AUTHOR CONTRIBUTIONS

The authors jointly conceived the study. JW led analyses and manuscript preparation. TB prepared datasets. DR developed theory. All authors contributed to manuscript preparation.

### PEER REVIEW

The peer review history for this article is available at https://publons.com/publon/10.1111/ele.13991.

## Supporting information

Supplementary Material

## Data Availability

All data associated with this study are publicly available on the Environmental Data Initiative repository (https://doi.org/10.6073/pasta/27e795dee803493140d6a7cdc3d23379). R scripts for reproducing this study are available on Zenodo (https://doi.org/10.5281/zenodo.6045931).
