# Supplementary material for: Tail‐dependent spatial synchrony arises from nonlinear driver–response relationships
Source: Ecol Lett. 2022 Mar 4;25(5):1189–201. doi: 10.1111/ele.13991 (PMC9543197; doi:10.1111/ele.13991)
Supplement: Supplementary file 1 — Supplementary Material [file ELE-25-1189-s001.pdf]

Supplementary Materials to: Tail-dependent spatial synchrony of giant kelp forests

Jonathan A. Walter[1], Max C. N. Castorani[1], Tom W. Bell[2], Lawrence W. Sheppard[3,4], Kyle C. Cavanaugh[5], and Daniel C. Reuman[3]

[1]Department of Environmental Sciences, University of Virginia
[2]Woods Hole Oceanographic Institution
[3]Department of Ecology and Evolutionary Biology and Center for Ecological Research and Kansas Biological Survey, University of Kansas
[4]Marine Biological Association of the United Kingdom
[5]Department of Geography, University of California, Los Angeles

S1 General theory

To substantiate our theoretical claim that, under fairly general conditions, appropriately nonlinear, synchronous environmental influences on population growth rates can produce tail dependent spatial synchrony (asymmetric tail associations) between population time series in different locations, we first formulate a general model. Suppose

$$N_i(t+1) = N_i(t)\lambda(N_i(t))\exp(e_s(t) + e_{l,i}(t)) \tag{S1}$$

for $i = 1, 2$, where $N_i(t)$ is population density at location i and time t, λ is a density-dependent growth rate, $e_s(t)$ is a spatially synchronous environmental effect on growth rates, and $e_{l,i}(t)$ represents local-noise effects on growth rates. We assume the $e_{l,i}(t)$ are normally distributed with mean $\overline{e}_l$ and standard deviation σ_l, and that they are independent across

both space and time. We use either and overbar or $\mathrm{E}(\cdot)$ to denote expected value of an expression. We assume the $e_s(t)$ are independent and identically distributed (iid) across time, and are independent of the $e_{l,i}(t)$.

We let $e_s(t) = f(\delta(t))$, where $\delta(t)$ is normal with mean $\overline{\delta}$ and standard deviation σ_s, and where

$$f(x) = \frac{c}{1 + \exp(-(\frac{x}{a} + b))} + d \tag{S2}$$

is a sigmoid function. The random variable $\delta(t)$ represents a spatially synchronous environmental variable and f represents its influence on population growth rates. The function f is shaped similarly to the logistic function, but it has been "stretched" both horizontally (controlled by the parameter a) and vertically (controlled by the parameter c), and also translated both horizontally (controlled by b) and vertically (controlled by d).

We assume $\lambda(N)$ is a monotonically decreasing continuous function of N, and that $\lim_{N\to\infty} \lambda(N) = 0$. Defining $\overline{e} = \overline{e}_s + \overline{e}_l$, we furthermore assume $\lambda(0) > 1/\exp(\overline{e})$. We can then uniquely define $N^* = \lambda^{-1}(1/\exp(\overline{e}))$, and note that $N^* = N^*\lambda(N^*)\exp(\overline{e}_s + \overline{e}_l)$, i.e., the deterministic one-patch model obtained by setting $e_s(t) = \overline{e}_s$ and $e_{l,i}(t) = \overline{e}_l$ has an equilibrium at N^*. We assume this equilibrium is stable, i.e.,

$$\left| \frac{d}{dN} \left[N\lambda(N) \exp(\overline{e}_s + \overline{e}_l) \right] \Big|_{N=N^*} \right| < 1. \tag{S3}$$

After some calculations (see next paragraph), this reduces to the equivalent assumption that

$$\frac{-2}{N^*} < \frac{\left(\frac{d\lambda}{dN} \big|_{N=N^*} \right)}{\lambda(N^*)}. \tag{S4}$$

This completes the presentation of the model and associated assumptions.

To see that (S4) is equivalent to (S3), we proceed as follows. First, by computing deriva-

tives, (S3) is equivalent to

$$\left|\lambda(N^*)\exp(\overline{e}) + N^* \frac{d\lambda}{dN}\bigg|_{N=N^*} \exp(\overline{e})\right| < 1. \tag{S5}$$

But we know from above that $N^* = N^*\lambda(N^*)\exp(\overline{e})$, so $1 = \lambda(N^*)\exp(\overline{e})$, so (S5) becomes

$$\left|1 + N^* \frac{d\lambda}{dN}\bigg|_{N=N^*} \exp(\overline{e})\right| < 1. \tag{S6}$$

This is equivalent to

$$0 < -N^* \frac{d\lambda}{dN}\bigg|_{N=N^*} \exp(\overline{e}) < 2. \tag{S7}$$

But $N^* > 0$, $\frac{d\lambda}{dN} < 0$, and $\exp(\overline{e}) > 0$, so the first inequality in (S7) is automatically satisfied, and therefore (S3) is equivalent to

$$N^* \frac{d\lambda}{dN}\bigg|_{N=N^*} \exp(\overline{e}) > -2. \tag{S8}$$

But because $\lambda(N^*) = 1/\exp(\overline{e})$ (see above), this is equivalent to (S4). The right side of (S4) is the instantaneous proportional change in $\lambda(N)$ per unit change in N, at $N = N^*$.

Model parameters are $\overline{e}_l$, σ_l, $\overline{\delta}$, σ_s, a, b, c, d, but some of these parameters turn out to be redundant. We now carry out simplifications and parameter reductions, starting with the observation that the parameters $\overline{\delta}$ and σ_s are redundant with the parameters a and b. Multiple combinations of these parameters can lead to the same distribution of $f(\delta)$, and the distribution of $f(\delta)$ is the only way these parameters influence model dynamics. It is straightforward to see that it suffices to set $a = 1$ and $b = 0$ – all distributions of $f(\delta)$ that can be obtained by utilizing full flexibility in the values of the parameters $\overline{\delta}$, σ_s, a, and b can also be obtained with $a = 1$ and $b = 0$.

Next, we claim that, without loss of generality, we can set $\overline{e}_l = 0$, and we can assume that

d takes a value such that $\mathrm{E}(f(\delta)) = 0$. To see this, define $\tilde{\lambda}(N) = \lambda(N)\exp(\mathrm{E}(f(\delta)) + \overline{e}_l)$, $\tilde{e}_s = e_s - \overline{e}_s = f(\delta) - \mathrm{E}(f(\delta))$, $\tilde{e}_{l,i} = e_{l,i} - \overline{e}_l$, and $\tilde{e} = \tilde{e}_s + \tilde{e}_{l,i}$. Then,

$$N_i(t+1) = N_i(t)\lambda(N_i(t))\exp(e_s(t) + e_{l,i}(t)) \tag{S9}$$

$$= N_i(t)\lambda(N_i(t))\exp(\tilde{e}_s(t) + \overline{e}_s + \tilde{e}_{l,i}(t) + \overline{e}_l) \tag{S10}$$

$$= N_i(t)\lambda(N_i(t))\exp(\overline{e}_s + \overline{e}_l)\exp(\tilde{e}_s(t) + \tilde{e}_{l,i}(t)) \tag{S11}$$

$$= N_i(t)\tilde{\lambda}(N_i(t))\exp(\tilde{e}_s(t) + \tilde{e}_{l,i}(t)). \tag{S12}$$

We then have $\overline{\tilde{e}}_{l,i} = 0$ and

$$\tilde{e}_s = e_s - \overline{e}_s \tag{S13}$$

$$= f(\delta) - \mathrm{E}(f(\delta)) \tag{S14}$$

$$= \frac{c}{1 + e^{-\delta}} + [d - \mathrm{E}(f(\delta))], \tag{S15}$$

which is equivalent to choosing d as described. The modified density-dependent growth rate, $\tilde{\lambda}(N)$, also satisfies the assumptions which were originally placed on $\lambda(N)$, i.e., $\tilde{\lambda}(N) = \lambda(N)\exp(\overline{e}_s + \overline{e}_l)$ is still obviously monotonically decreasing and continuous, and satisfies $\lim_{N\to\infty}\tilde{\lambda}(N) = 0$. And we have $\tilde{\lambda}(0) > 1/\exp(\overline{\tilde{e}}) = 1$, since $\tilde{\lambda}(0) = \lambda(0)\exp(\overline{e}) > \frac{1}{\exp(\overline{e})}\exp(\overline{e}) = 1$. Furthermore, because $\tilde{\lambda}(0) > 1 = 1/\exp(\overline{\tilde{e}})$, we can uniquely define $\tilde{N}^* = \tilde{\lambda}^{-1}(1/\exp(\overline{\tilde{e}})) = \tilde{\lambda}^{-1}(1)$, an equilibrium of the deterministic skeleton of the new model, (S12). We want to show this is a stable equilibrium, i.e.,

$$\left| \frac{d}{dN}[N\tilde{\lambda}(N)\exp(\overline{\tilde{e}})]\Big|_{N=\tilde{N}^*} \right| < 1. \tag{S16}$$

But $\frac{d}{dN}[N\tilde{\lambda}(N)\exp(\overline{\tilde{e}})] = \frac{d}{dN}[N\lambda(N)\exp(\overline{e}_s + \overline{e}_l)]$, so (S16) reduces to (S3), which was already satisfied, by the assumptions placed on the original model.

Therefore, after parameter reduction, it suffices to consider a model (S1) where the following assumptions are met:

- The $e_{l,i}(t)$ are normally distributed with mean 0 and standard deviation σ_l and are iid across space and time.
- The synchronous environmental driver, $e_s(t)$ is of the form $e_s(t) = \frac{c}{1+e^{-\delta(t)}} + d$. Here the $\delta(t)$ are independent of the $e_{l,i}(t)$ and are iid through time, and are normally distributed with mean $\overline{\delta}$ and standard deviation σ_s. And d is selected so that $\overline{e}_s = 0$.
- The density-dependent growth rate, $\lambda(N)$, is a monotonically decreasing, continuous function of N such that $\lim_{N\to\infty} \lambda(N) = 0$, and such that $\lambda(0) > 1$. Furthermore, the model equilibrium is stable, which amounts to assuming $\frac{d\lambda}{dN}\big|_{N=N^*} > \frac{-2}{N^*}$ for $N^* = \lambda^{-1}(1)$.

The free parameters of the model are then σ_l, c, $\overline{\delta}$, and σ_s. The functional form of λ is also flexible, subject to the listed assumptions/constraints; though we will find, below, by linearizing, that for stochasticity of modest intensity, only certain features of λ make a difference.

We next linearize the model. Letting $g(N, e_s, e_l) = N\lambda(N)\exp(e_s + e_l)$ and $x_i(t) = N_i(t) - N^*$, we have

$$x_i(t+1) \approx \frac{\partial g}{\partial N}\bigg|_{(N^*,0,0)} x_i(t) + \frac{\partial g}{\partial e_s}\bigg|_{(N^*,0,0)} e_s(t) + \frac{\partial g}{\partial e_l}\bigg|_{(N^*,0,0)} e_{l,i}(t). \tag{S17}$$

Here,

$$\frac{\partial g}{\partial N} = \left(\lambda(N) + N\frac{d\lambda}{dN}\right)\exp(e_s + e_l) \tag{S18}$$

$$\left.\frac{\partial g}{\partial N}\right|_{(N^*,0,0)} = \lambda(N^*) + N^*\left.\frac{d\lambda}{dN}\right|_{N=N^*} \tag{S19}$$

$$= 1 + N^*\left.\frac{d\lambda}{dN}\right|_{N=N^*} \tag{S20}$$

$$\frac{\partial g}{\partial e_s} = N\lambda(N)\exp(e_s + e_l) = g \tag{S21}$$

$$\left.\frac{\partial g}{\partial e_s}\right|_{(N^*,0,0)} = N^* \tag{S22}$$

$$\frac{\partial g}{\partial e_l} = N\lambda(N)\exp(e_s + e_l) = g \tag{S23}$$

$$\left.\frac{\partial g}{\partial e_l}\right|_{(N^*,0,0)} = N^*. \tag{S24}$$

Therefore, (S17) becomes

$$x_i(t+1) \approx Ax_i(t) + Be_s(t) + Be_{l,i}(t), \tag{S25}$$

where

$$A = 1 + N^*\left.\frac{d\lambda}{dN}\right|_{N=N^*} \tag{S26}$$

$$= 1 + \lambda^{-1}(1)\left.\frac{d\lambda}{dN}\right|_{N=N^*} \tag{S27}$$

$$B = N^* = \lambda^{-1}(1). \tag{S28}$$

We know $-1 < A < 1$, by model assumptions, so this linearized model is a stable ARMA-like process of autoregressive order 1. Any solution $x_i(t)$ to (S25) is paired with a solution

$x_i(t)/B$ to the modified model

$$x_i(t+1) \approx Ax_i(t) + e_s(t) + e_{l,i}(t), \tag{S29}$$

and the two solutions will have the same synchrony and tail association properties, so it suffices to study (S29). For similar reasons, we can assume, without loss of generality, that $c = 1$. For "weak noise" (i.e., sufficiently small standard deviations of $e_s(t)$ and $e_{l,i}(t)$), the dynamics of (S29) are essentially the same, for our purposes, as the dynamics of the original model. So we henceforth consider (S29), which has only the parameters $-1 < A < 1$, $\sigma_l > 0$, $\overline{\delta}$, and $\sigma_s > 0$. We next perform a simulation study to see if outputs of (S29) show asymmetric tail association, and how that depends on model parameters.

For all possible combinations of $A = -0.8, -0.5, -0.3, 0, 0.3, 0.5, 0.8$, $\sigma_l = 0.1, 0.2, 0.3$, $\overline{\delta} = -8, -7.9, -7.8, \cdots, 7.8, 7.9, 8$, and $\sigma_s = 0.5, 0.6, 0.7, \cdots, 2.9, 3$, we simulated the model (S29) starting from $x_i(0) = 0$, discarded 100 time steps of model "burn-in" to eliminate any transient dynamics, and retained the following 1000 time steps of model simulation results. We then computed the partial Spearman correlation of the results, across habitat patches, using bounds $b_l = 0.5$ and $b_u = 1$; this quantifies upper-tail association, where the notation b_l, b_u follows Ghosh *et al.* (2021). We then computed the partial Spearman correlation of the simulation results using bounds $b_l = 0$ and $b_u = 0.5$. This quantifies lower-tail association. We then computed the upper-tail-association value minus the lower-tail-association value. This difference quantifies asymmetry of tail association, with positive values indicating greater upper-tail association. For each combination of $A = -0.8, -0.5, -0.3, 0, 0.3, 0.5, 0.8$ and $\sigma_l = 0.1, 0.2, 0.3$, we plotted this difference against $\overline{\delta}$ and σ_s (Fig. S1).

Results showed clearly that when the distribution of δ substantially overlapped the left "shoulder" of the logistic sigmoid, resulting populations were predominantly upper-tail associated; whereas when the distribution of δ substantially overlapped the right "shoulder"

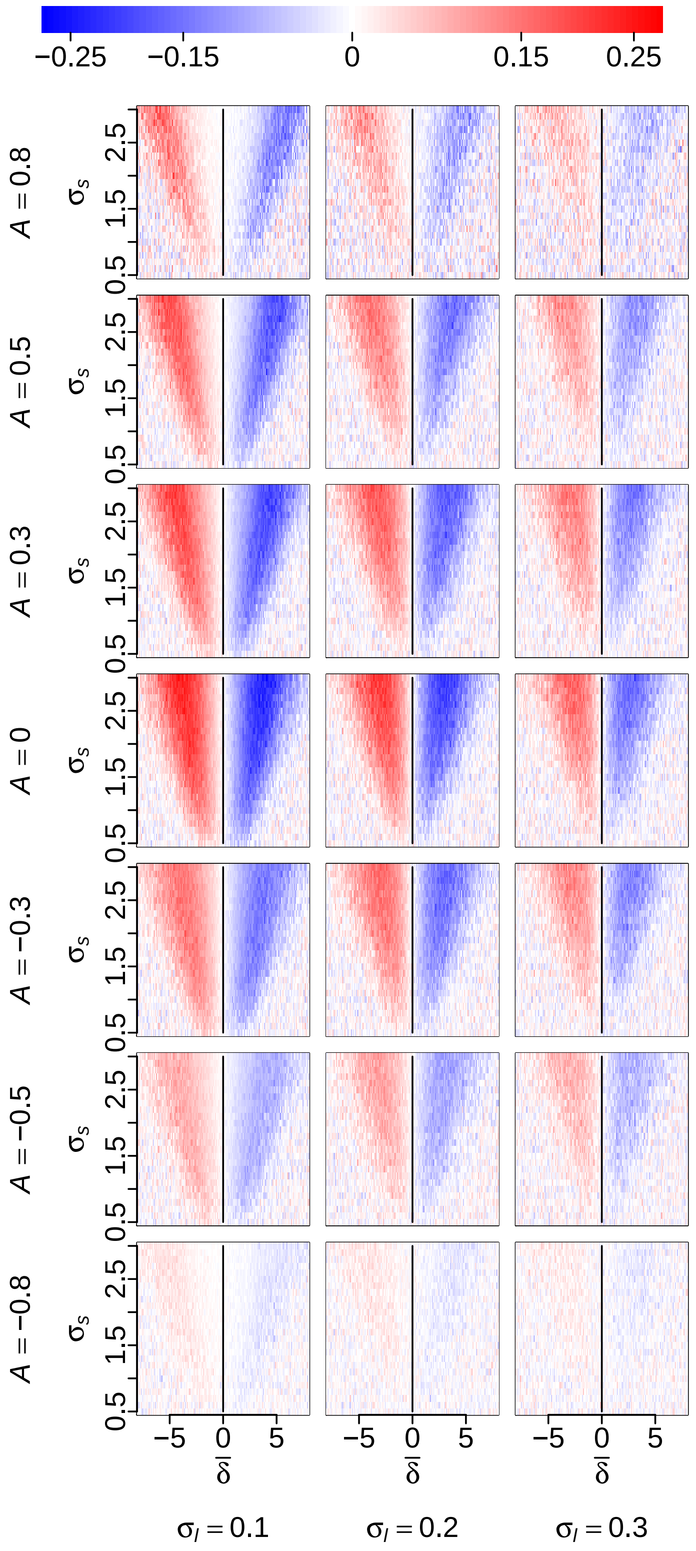

Figure S1: Results of the simulation study in section S1. See text for details.

of the logistic sigmoid, populations were predominantly lower-tail associated. These patterns were mitigated for strong density dependence (i.e., large $|A|$), especially for strongly over-compensatory dynamics (i.e., strongly negative A), but were detectable even in those cases.

S2 Example of the theory

Figure 2 of the main text was produced using (S29) with $A = 0.5$, $\sigma_l = 0.15$, $\overline{\delta} = 2$ or -2, in the two scenarios considered, and $\sigma_s = 1.5$. Simulations for Figure 2 were run for 150 time steps, discarding the first 50 time steps to eliminate effects of initial conditions.

S3 Formal definition of the partial Spearman correlation

Given two variables, here indexed by time, $x(t)$ and $y(t)$, for $t = 1, \ldots, T$, we first define the *normalized rank* $u(t)$, which equals the rank of $x(t)$ in the set $\{x(1), \ldots, x(T)\}$, divided by $T + 1$. The smallest member of this set is here considered to have rank 1. We likewise define the normalize rank $v(t)$, which is the rank of $y(t)$ in the set $\{y(1), \ldots, y(T)\}$, divided by $T + 1$. Plotting u and v against each other provides a visual indication of asymmetries of tail association (Ghosh *et al.*, 2020a,b,c, 2021); points of this plot are all within the unit square $[0, 1] \times [0, 1]$. Given the bounds $0 \leq b_l < b_u \leq 1$, we define the lines $u + v = 2b_l$ and $u + v = 2b_u$, which intersect the unit square. The partial Spearman correlation associated with the band circumscribed by these lines is

$$\mathrm{cor}_{b_l,b_u}(x, y) = \frac{\sum(u(t) - \mathrm{mean}(u))(v(t) - \mathrm{mean}(v))}{(T - 1)\sqrt{\mathrm{var}(u)\,\mathrm{var}(v)}}, \tag{S30}$$

where sample variances and means are computed over $t = 1, \ldots, T$ but the summation is only computed over t such that $u(t) + v(t) > 2b_l$ and $u(t) + v(t) < 2b_u$. These are the t corresponding to the points in the band described above.

S4 Details of spatial linear regression and model assumptions

To test for statistical effects on tail dependence in giant kelp spatial synchrony of tail dependence in the relationship between kelp biomass and, respectively, wave calmness, the NPGO, and seawater nitrate concentration, we used multiple regression fit using generalized least squares (Pinheiro & Bates, 2000). The generalized least squares framework has similar assumptions to ordinary least squares regression, including linearity of relationships between predictor and response, and normality of residuals, but allows for various functional forms of correlation in the residuals. Using the 'gls' function in the 'nlme' R package, we fit multiple regression models with the error term having spatial correlation that decays exponentially with distance. The model is:

$$y = \beta_0 + \beta_1 x_1 + \beta_2 x_2 + \beta_3 x_3 + \epsilon, \mathrm{Cov}(\epsilon) = a \times \exp(-d/b). \tag{S31}$$

Here, y is the tail dependence strength of giant kelp synchrony averaged over all sites within some threshold distance of the central site (we consider a threshold of 25 km in the main text; see tables S1-S4 for additional thresholds from 10 to 200 km). The β_n are regression parameters including the intercept. The x_n are the predictors tail dependence strength in, respectively, wave calmness, the NPGO, and seawater nitrate concentration; these are also averaged over the same distance thresholds as tail dependence strength of giant kelp synchrony. ϵ is the error term, the spatial covariance of which is specified to decay exponentially

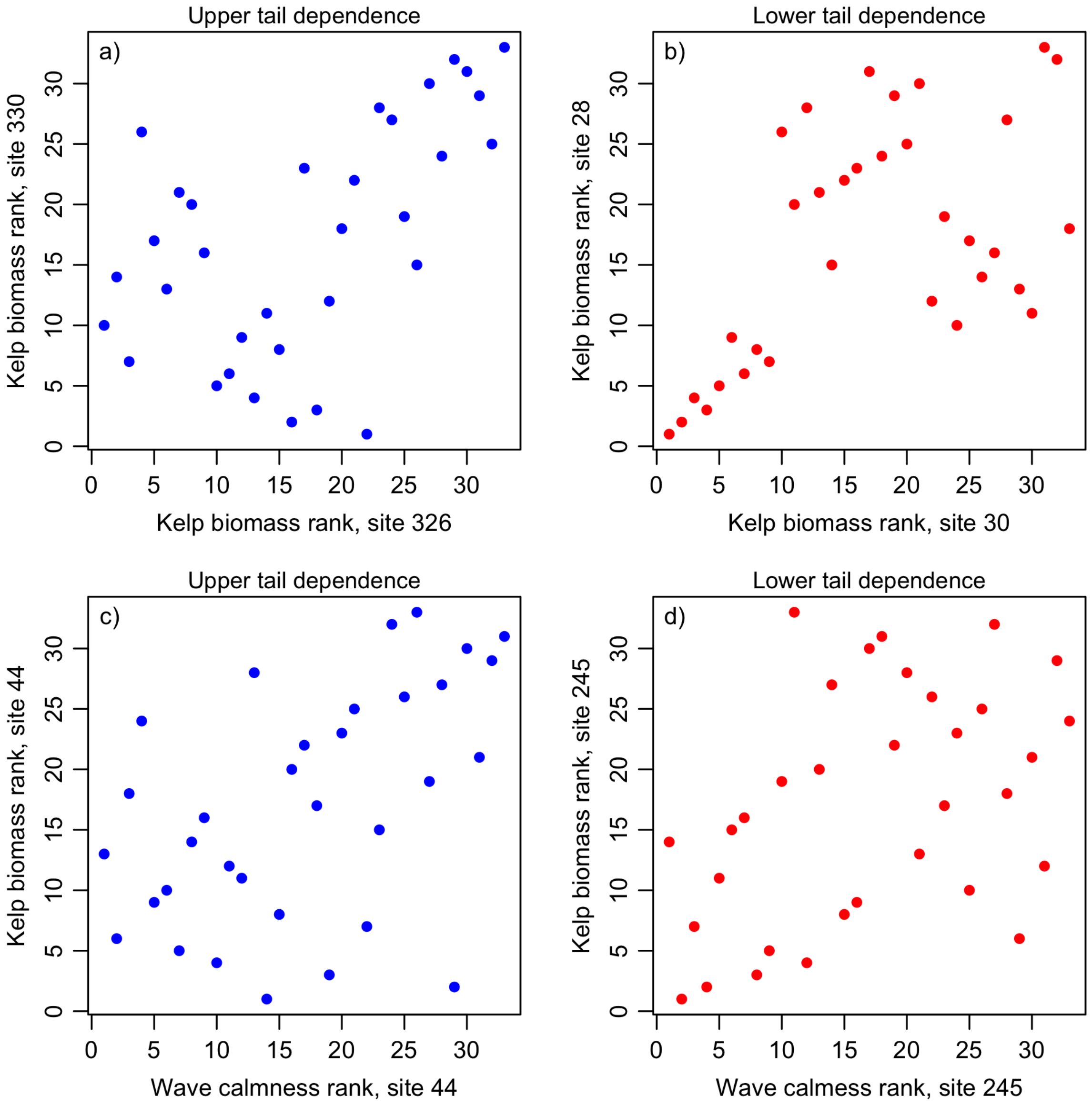

Figure S2: Examples of tail association in empirical data analogous to theoretical illustrations in Figure 2b-e.

Parameter	Estimate	Std. Err.	p-value
Intercept	0.0005	0.0008	0.5176
Nitrate	-0.021	0.037	0.5848
Wave calmness	0.067	0.030	0.0283
NPGO	0.0092	0.035	0.7930

Table S1: Spatial multiple linear regression relating tail dependence between giant kelp biomass and driver variables to tail dependence in giant kelp spatial synchrony. The distance threshold for averaging was 10 km.

with distance d; a is the nugget and b is the range.

We tested whether model assumptions were met by examining normal QQ plots of model residuals and asking whether spatial autocorrelation in model residuals approximately follows a negative exponential distribution. Spatial autocorrelation was examined using spline correlograms (Bjørnstad & Falck, 2001) using the 'ncf' package in R. The assumption of normality of residuals was well-met by the statistical models (see, e.g., Figure S3a), but spatial autocorrelation in model residuals was not particularly well-described by a negative exponential function (e.g., Figure S3b). However, we proceeded using this model for reasons enumerated below. The observed relationship between spatial autocorrelation and distance is non-monotonic, but existing approaches including other correlation structures available in 'nlme' and notable alternatives like spatial lag models assume monotonic distance-decay in autocorrelation. Hence, there is no clear optimal solution, but by accounting–even imperfectly–for spatial autocorrelation, our models are more conservative than ordinary least squares regression.

We used the same procedures to evaluate the relationship between tail dependence in kelp spatial synchrony and site mean wave calmness. Similarly, model residuals were consistent with the assumption of normality of residuals, but distance-decay of spatial autocorrelation was non-monotonic but we proceeded with using a negative exponential correlation for the error term for the same reasons as above.

Parameter	Estimate	Std. Err.	p-value
Intercept	0.0011	0.0010	0.3084
Nitrate	-0.015	0.031	0.6305
Wave calmness	0.099	0.026	0.0002
NPGO	-0.0069	0.028	0.8085

Table S2: Spatial multiple linear regression relating tail dependence between giant kelp biomass and driver variables to tail dependence in giant kelp spatial synchrony. The distance threshold for averaging was 25 km; these results are also reported in the main text.

Parameter	Estimate	Std. Err.	p-value
Intercept	0.0021	0.0010	0.0469
Nitrate	0.016	0.027	0.5648
Wave calmness	0.098	0.023	< 0.0001
NPGO	-0.020	0.026	0.4474

Table S3: Spatial multiple linear regression relating tail dependence between giant kelp biomass and driver variables to tail dependence in giant kelp spatial synchrony. The distance threshold for averaging was 50 km.

Parameter	Estimate	Std. Err.	p-value
Intercept	0.0042	0.0025	0.0886
Nitrate	0.017	0.025	0.5032
Wave calmness	0.098	0.021	< 0.0001
NPGO	0.013	0.025	0.5741

Table S4: Spatial multiple linear regression relating tail dependence between giant kelp biomass and driver variables to tail dependence in giant kelp spatial synchrony. The distance threshold for averaging was 100 km.

Parameter	Estimate	Std. Err.	p-value
Intercept	0.00074	0.0035	0.8332
Nitrate	0.0099	0.025	0.6917
Wave calmness	0.11	0.021	< 0.0001
NPGO	0.036	0.023	0.51322

Table S5: Spatial multiple linear regression relating tail dependence between giant kelp biomass and driver variables to tail dependence in giant kelp spatial synchrony. The distance threshold for averaging was 200 km.

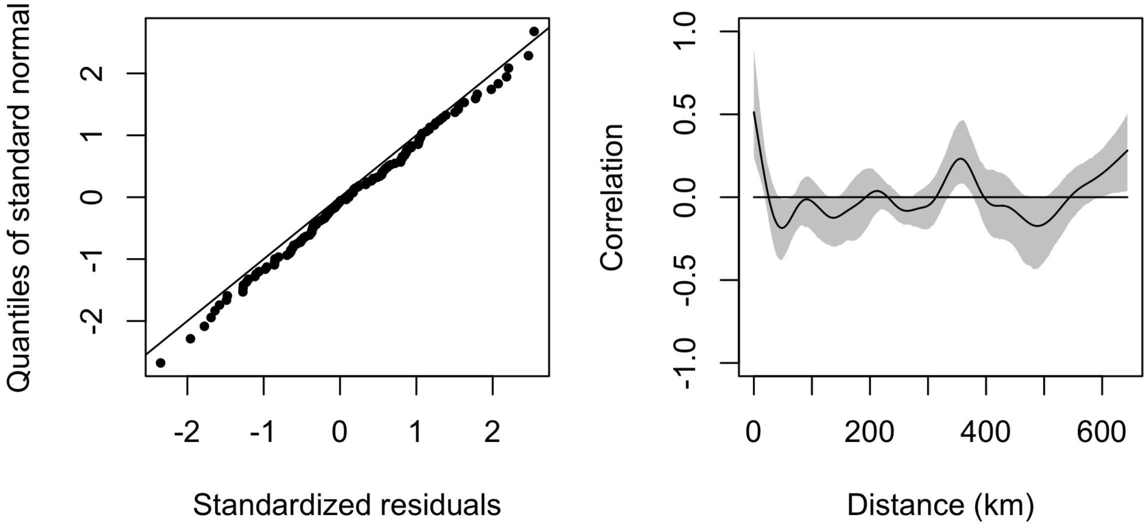

Figure S3: Regression diagnostics for a model of tail dependence in giant kelp spatial synchrony as a function of tail dependence in the relationship between giant kelp abundance and, respectively, wave calmness, the NGPO, and seawater nitrate concentration. The distance threshold for averaging was 25 km. a) normal QQ plot. b) spline correlogram showing spatial autocorrelation as a function of distance.

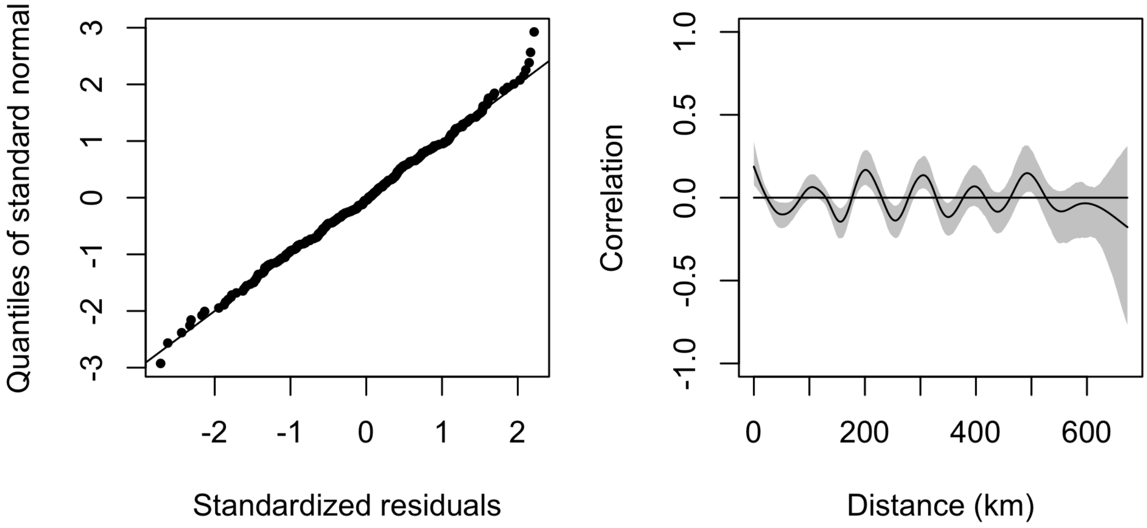

Figure S4: Regression diagnostics for a model of tail dependence in giant kelp spatial synchrony as a function of site mean wave calmness. The distance threshold for averaging was 25 km. a) normal QQ plot. b) spline correlogram showing spatial autocorrelation as a function of distance.

References

Bjørnstad, O. N. & Falck, W. (2001). Nonparametric spatial covariance functions: estimation and testing. *Environmental and Ecological Statistics*, 8, 53–70.

Ghosh, S., Cottingham, K. L. & Reuman, D. C. (2021). Species relationships in the extremes and their influence on community stability. *Philosophical Transactions of the Royal Society B*, 20200343.

Ghosh, S., Sheppard, L. W., Holder, M. T., Loecke, T. D., Reid, P. C., Bever, J. D. & Reuman, D. C. (2020a). Chapter eleven - copulas and their potential for ecology. In: *Tropical Ecosystems in the 21st Century* (eds. Dumbrell, A. J., Turner, E. C. & Fayle, T. M.), vol. 62 of *Advances in Ecological Research*. Academic Press, pp. 409–468. URL `https://www.sciencedirect.com/science/article/pii/S0065250420300039`.

Ghosh, S., Sheppard, L. W., Reid, P. C. & Reuman, D. C. (2020b). A new approach to interspecific synchrony in population ecology using tail association. *Ecology and Evolution*, 10, 12764–12776.

Ghosh, S., Sheppard, L. W. & Reuman, D. C. (2020c). Tail associations in ecological variables and their impact on extinction risk. *Ecosphere*, 11, e03132.

Pinheiro, J. C. & Bates, D. M. (2000). *Linear mixed-effects models: basic concepts and examples*. Springer.
